# Assessing the Impact of IL-6 and Serotonin on Pain and Symptomatology in Fibromyalgia: An Exploratory Clinical Study

**DOI:** 10.3390/jpm14080886

**Published:** 2024-08-22

**Authors:** Felipe Altino Loçasso, Hélcio Alvarenga Filho, Regina Maria Papais Alvarenga, Sérgio Luís Schimidt, Filipe Kleinman Fiorelli, Plínio dos Santos Ramos, Sônia Cristina Leal Leidersnaider, Kenneth Blum, Kai-Uwe Lewandrowski, Edezio Ferreira Cunha-Junior, Rossano Kepler Alvim Fiorelli

**Affiliations:** 1 Programa de Pós-Graduação em Neurologia, Universidade Federal do Estado do Rio de Janeiro, Rio de Janeiro 20270-004, Brazil; helcio_alvarenga@hotmail.com (H.A.F.); regina_alvarenga2004@hotmail.com (R.M.P.A.); slschmidt@terra.com.br (S.L.S.); fiorellirossano@hotmail.com (R.K.A.F.); 2 Faculdade de Ciências Médicas de Três Rios (SUPREMA), Rio de Janeiro 25804-250, Brazil; filipe.fiorelli@aluno.suprema.edu.br (F.K.F.); pliniosramos@suprema.edu.br (P.d.S.R.); sonia.leidersnaider@tr.suprema.edu.br (S.C.L.L.); 3 Division of Addiction Research & Education, Center for Sports, Exercise & Mental Health, Western University, Health Sciences, Pomona, CA 91766, USA; drd2gene@gmail.com; 4 Center for Advanced Spine Care of Southern Arizona, The Surgical Institute of Tucson, 4787 E Camp Lowell Dr., Tucson, AZ 85712, USA; business@tucsonspine.com; 5 Instituto de Ciências Farmacêuticas, Centro Multidisciplinar UFRJ-Macaé, Universidade Federal do Rio de Janeiro, Rio de Janeiro 27930-560, Brazil; edezio@macae.ufrj.br

**Keywords:** fibromyalgia, FIQ-R, IL-6 and serotonin

## Abstract

Background and Objectives: Fibromyalgia (FM) is a chronic musculoskeletal pain syndrome characterized by widespread pain and a variety of other symptoms, including fatigue, cognitive dysfunction, and sleep disturbances. Recent research has highlighted the potential role of pro-inflammatory cytokines and neurotransmitters in the pathophysiology of FM. This study aimed to investigate the relationship between serum levels of interleukin-6 (IL-6) and serotonin with the clinical parameters observed in patients with fibromyalgia. Additionally, it sought to analyze the similarities and differences among the different groups classified by symptom severity. Materials and Methods: This cross-sectional study included 26 female patients aged 20–70 diagnosed with FM according to the American College of Rheumatology (ACR) 2016 criteria and 14 healthy controls (HCs). Serum levels of IL-6 and serotonin were measured using electrochemiluminescence and high-performance liquid chromatography (HPLC), respectively. Results: FM patients exhibited significantly higher pain scores (VAS), anxiety, and depression levels compared to HCs. FIQ-R scores were significantly elevated in FM patients, with stratification showing 3.8% mild, 65.4% moderate, 23.1% severe, and 7.7% very severe cases. While no significant difference in IL-6 levels was observed between the FM patients and HCs, a trend towards increased IL-6 levels in patients with higher FIQ-R scores was noted. Serum serotonin levels were significantly lower in the FM patients than in the HCs, with moderate patients having lower levels than those classified as severe and very severe. Conclusions: The study underscores the potential role of IL-6 and serotonin in the pathophysiology of FM, suggesting that these biomarkers could be relevant in assessing the severity and impact of FM. Further research is needed to elucidate these relationships and their implications for developing personalized treatment strategies.

## 1. Introduction

Fibromyalgia (FM) is a chronic musculoskeletal pain syndrome characterized by diffuse pain associated with several other symptoms, such as fatigue, cognitive changes, and changes in sleep patterns [1]. It affects around 1 to 8% of the world population and 2% of the Brazilian population, with a predominance of females. The reasons for this predominance may include (1) biological factors, (2) genetic predisposition, (3) psychological and social factors, (4) healthcare-seeking behavior, and (5) socioeconomic factors (Table 1). Among rheumatological pathologies, FM is the third most common diagnosis in rheumatology outpatient clinics [2,3]. The etiology of fibromyalgia remains unknown. However, we know that the pathophysiology of FM involves a dysfunction of neurocircuits, with an imbalance of neurotransmitters generating a hyperfunctional state of neurons and nociceptive pathways caused by membrane hyperexcitability or reduced inhibition [4]. In this context, an increase in excitatory neurotransmitters (glutamate and substance P) is found as well as a reduction in neurotransmitters of the descending pain inhibitory pathway (serotonin and noradrenaline). Therefore, considering that central sensitization is a notable factor for these patients, other factors, such as genetic, immunological, and hormonal factors, must also participate in this pathway [1,5].

Recent forays into the neuroimmunology of FM have highlighted the crucial importance of serum serotonin, a central neurotransmitter with a diverse range of neuromodulatory functions. Amid the myriad of neurotransmitter systems implicated in pain and mood regulation, serotonin emerges as a centerpiece, the imbalance of which can trigger and perpetuate the debilitating symptoms associated with FM. The relationship between serotonin and FM is complex and not yet completely understood. Some researchers suggest that serotonin deficiency may lead to a reduced ability to modulate pain, resulting in greater pain sensitivity in FM patients. Additionally, serotonin also plays a role in regulating sleep, and sleep disturbances are common among people with FM [11]. Although fibromyalgia is currently not considered an inflammatory disease, we also know that pro-inflammatory mediators are present in FM patients [12,13]. Within the profile of pro-inflammatory cytokines, IL-6 and IL-8 stand out, which play the role of modulating responses in the sympathetic nervous system and the hypothalamic–pituitary–adrenal axis [14]. In recent years, research has highlighted the importance of serum serotonin and interleukin-6 (IL-6) in the pathophysiological mechanisms of FM. In this complex scenario, the need for a holistic approach is clear—one that addresses not only symptomatic manifestations but also the interplay of biochemical, genetic, neurobiological, and psychosocial factors.

This work aims to explore the relationship between serum levels of IL-6 and serotonin and the results of the Fibromyalgia Impact Questionnaire (FIQR [15]; Table 2), the Severity Score, and the Visual Analog Pain Scale (VAS) [16], fundamental tools in assessing the severity and functional impact of this complex syndrome. Furthermore, we aspire to provide insights for clinical practice as well as create opportunities for more effective therapies and personalized approaches to treating this debilitating condition.

## 2. Materials and Methods

To answer the research question, we designed a cross-sectional study comparing FMS patients with healthy controls (HCs). All procedures were carried out with the written informed consent of the subjects. This study was approved by the Research Ethics Committee of the Faculty of Medical and Health Sciences of Juiz de Fora-CEP/FCMS-JF (Code 5.592.482 and CAAE: 58101222500005103). All participants signed an informed consent form before the study in accordance with the Declaration of Helsinki and World Health Organization standards for observational studies. This study was carried out from 2023 to 2024.

### 2.1. Study Design

To confirm individual eligibility, all potential participants were asked to complete a brief questionnaire about their health condition and were subjected to a standardized clinical examination to confirm the diagnosis of fibromyalgia. The diagnostic criteria for fibromyalgia included generalized pain, defined as (1) pain in at least 4 out of 5 regions, along with a (2) Widespread Pain Index (WPI) score of ≥7 and a Symptom Severity Scale (SSS) score of ≥5, or a WPI score of 4–6 and an SSS score of ≥9, and (3) symptoms that have been present at similar levels for at least 3 months. From the moment of diagnosis, patients were invited to take part in this project. Patients treated at the rheumatology outpatient clinic of the Walter Gomes Francklin Polyclinic (CNES 2293749) located in the city of Três Rios, Rio de Janeiro, through a partnership with the Municipal Health Department of Três Rios, RJ, were selected.

Inclusion criteria: Women of Brazilian nationality aged between 20 and 70 years old and diagnosed with fibromyalgia were selected for inclusion in this study, which adhered to the criteria of the American College of Rheumatology (ACR) 2016.

Exclusion criteria: The exclusion criteria were presence of any type of immune mediated inflammatory joint disease, according to previous medical evaluation.

A total of 27 fibromyalgia patients and 16 control patients were recruited for the study. After applying the exclusion criteria, one patient from the fibromyalgia group was removed due to a diagnosis of immune-mediated inflammatory disease, and two patients from the control group were removed because they presented with inflammatory symptoms during the examination.

FM patients: After applying the inclusion and exclusion criteria, we selected 26 patients for inclusion in the FM group.

Healthy Control (HC): A total of 14 female HCs aged between 20 and 70 years were recruited and selected. Exclusion criteria were having chronic pain, being pregnant, and currently breastfeeding.

Clinical Measures: Comprehensive clinical assessments were performed using self-administered questionnaires and physical examinations. Self-reported clinical measures included the following assessments. (1) Visual Analogue Scale (VAS): This is a 10 cm linear scale ranging from 0 (no pain) to 10 (severe, unbearable pain). (2) Fibromyalgia Impact Questionnaire (FIQ-R): This questionnaire assesses the impact of fibromyalgia on patients’ lives across three domains: general impact, function, and symptoms. (3) Hospital Anxiety and Depression Scale (HADS): This tool aims to detect mild degrees of affective disorders and consists of fourteen multiple-choice items, with seven items assessing anxiety (HADS-A) and seven assessing depression (HADS-D). Each item is scored from 0 to 3, with a maximum of 21 points in each subscale. In this study, the literature-reported cut-off points were adopted: an anxiety score equal to or greater than 8, and a depression score equal to or greater than 9. (4) Widespread Pain Index (WPI): This index notes the number of areas where the patient has experienced pain over the past week, with a score ranging from 0 to 19. (5) Symptom Severity Scale (SSS): This scale sums the severity scores of three symptoms (fatigue, waking unrefreshed, and cognitive symptoms) (0–9) and the sum (0–3) of the number of additional symptoms a patient has experienced in the previous six months. The final symptom severity score ranges from 0 to 12. (6) Fibromyalgia Severity (FS) Scale: This scale is the sum of the WPI and SSS scores.

### 2.2. IL-6 and Serotonin Measures

Peripheral venous blood samples were drawn immediately into tubes without anticoagulant on the day of admission for the determination of serotonin and IL-6 levels. The blood samples were centrifuged at 2500 rpm for 10 min, and the sera were separated for the determination of IL-6 levels using the electrochemiluminescence technique and serotonin levels using high-performance liquid chromatography (HPLC).

### 2.3. Statistics Analysis

Statistical analysis was performed in GraphPad Prism (version 9.0). To evaluate differences between groups, unpaired Student’s t-test was used, and the significance level was established at a *p* value ≤ 0.05.

## 3. Results

### 3.1. Characteristics of HCs and FMS Patients

The ages of the patients with FMS and the HCs were not significantly different (Table 3). However, as expected, other clinical characteristics, such as pain scores (VAS), anxiety, and depression (with the last two corresponding to HADS-A and HADS-D, respectively), were significantly higher among the FMS patients compared to the HCs.

**Figure 1:**
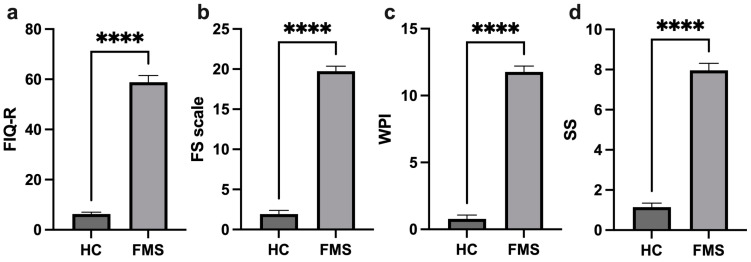
Comparison of profile clinical parameters of the healthy controls and FMS patients. FIQ-R (Revised Fibromyalgia Impact Questionnaire) (**a**), FS (Fibromyalgia Severity) scale (**b**), WPI (Widespread Pain Index) (**c**), and SS (Symptom Severity) scale (**d**), ****: *p* < 0.0001.

The assessments applied, such as the FIQ-R (Revised Fibromyalgia Impact Questionnaire), SS (symptom severity) scale, WPI (widespread pain index), and FS (fibromyalgia severity) scale, demonstrate the significant difference in these clinical parameters between the patients with FMS and the HCs, with high scores among the FMS patients (Figure 1). The patients with FMS were stratified into groups according to the severity indicated by the FIQ-R: 3.8% mild, 65.4% moderate, 23.1% severe, and 7.7% very severe.

### 3.2. Levels of the IL-6 and Serotonin in Healthy Controls and FMS Patients

**Figure 2:**
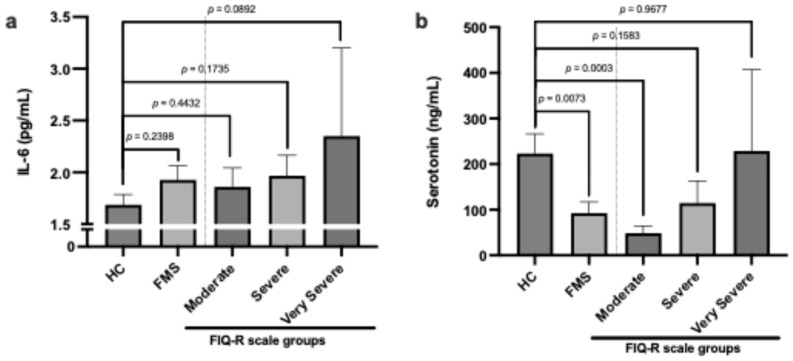
Comparison of levels of IL-6 (**a**) and serotonin (**b**) in healthy controls and FMS patients with FIQ-R scale classification.

Patients with FMS showed a tendency towards increased IL-6 levels, although the difference was not significant. Interestingly, IL-6 levels were slightly higher in patients according to group stratification by FIQ-R (Figure 2a). Regarding serum serotonin levels, we found that patients with FMS had lower levels compared to HC. However, by stratifying the groups according to the FIQ-R classification, we observed that the greater the severity, the higher the serotonin levels (Figure 2b).

## 4. Discussion

The interrelationship between fibromyalgia and inflammatory biomarkers, such as IL-6, IL-8, and TNF-α, has been the subject of an increasing number of studies. IL-6 is a pro-inflammatory cytokine produced by a variety of cells, including leukocytes and muscle cells, and plays a central role in regulating immune responses and mediating chronic inflammation. IL-6 is associated with central sensitization, thus exacerbating the pain experienced by FM patients [17,18]. Although the causal relationship between IL-6 and fibromyalgia has not been fully elucidated, there is evidence that systemic inflammation and immune system activation may play a role in the pathogenesis and clinical manifestations of the disease [19]. Therapeutic strategies that aim to modulate the inflammatory response, including the use of anti-inflammatory and immunomodulatory agents, represent a promising area of research in the treatment of fibromyalgia [20,21]. The relationship between FIQ-R scores and interleukin 6 (IL-6) levels offers additional insights into the mechanisms underlying fibromyalgia symptomatology. Studies have demonstrated an association between higher FIQ-R scores and increased IL-6 levels in patients with fibromyalgia. This association suggests that the severity of patient-reported symptoms, as assessed by the FIQ-R, may be related to immune system activation and the presence of systemic inflammation, as indicated by IL-6 and IL-8 [14]. Although in our work we did not observe a significant difference in IL-6 levels between the FMS and HC patients (Figure 2a), the trend towards increased IL-6 levels in patients with a higher FIQ-R score corroborates the data reported by Mendieta et al. [14]. IL-6 may play a central role in modulating many of the symptoms assessed by the FIQ-R. For example, IL-6 is implicated in sleep regulation, stress responses, and fatigue, all of which are domains addressed by FIQ-R. Furthermore, pro-inflammatory cytokines, such as IL-1 and IL-6, play a role in pain modulation through specific mechanisms. IL-1β enhances excitatory currents within the spinal cord, while both IL-1β and IL-6 suppress inhibitory currents induced by GABA and glycine. This results in a feed-forward cycle of nociceptive signaling, ultimately leading to central sensitization [19].

The relationship between serotonin and fibromyalgia is complex and not yet completely understood. Considering the preponderant role of serotonin in regulating pain sensitivity and pain processing in the central nervous system, as well as mood and sleep, many studies have found reduced levels of serotonin in patients with fibromyalgia, suggesting that this deficiency may be associated with some symptoms of this syndrome. Depression and anxiety are often associated with fibromyalgia, and low serotonin may contribute to these symptoms, exacerbating patients’ pain perception and quality of life [22]. Serotonin’s relationship with the FIQ-R has been the subject of growing interest in the scientific community. Previous observations suggested an inverse correlation between serum serotonin levels and FIQ-R scores, implying a possible contribution of this neurotransmitter to the manifestation and severity of FM symptoms [11]. Our data show that there are lower serum serotonin levels in FMS patients compared to HCs, with moderate patients having lower levels than severe and very severe patients (Figure 2b). Al-Nimer et al. indicated in their study that serum serotonin levels would not be a useful predictor of severity considering clinical symptoms assessed using FIQ-R scores. But women recently diagnosed with FM had low levels of serotonin [23].

## 5. Conclusions

This study underscores the potential role of IL-6 and serotonin in the pathophysiology of FM, suggesting that these biomarkers could be relevant in assessing the severity and impact of FM. Further research is needed to elucidate these relationships and their implications for developing personalized treatment strategies.

## Figures and Tables

**Table 1:** Contributing factors for the higher prevalence of fibromyalgia in women compared to men.

Factor	Description
Biological Factors	Hormonal differences between men and women, such as estrogen levels, may influence pain perception and sensitivity to pain, potentially contributing to the development or exacerbation of fibromyalgia symptoms [6].
Genetic Predisposition	Genetic factors may predispose women more than men to developing fibromyalgia. Familial aggregation of fibromyalgia suggests there is a genetic component to this disorder [7].
Psychological and Social Factors	Women may experience different stressors, coping mechanisms, and societal pressures compared to men, which could contribute to the onset or exacerbation of fibromyalgia symptoms. Psychological factors like stress, anxiety, and depression can influence pain perception and increase the risk of developing fibromyalgia [8].
Healthcare-Seeking Behavior	Women tend to seek healthcare more frequently than men, potentially leading to higher rates of diagnosis. Fibromyalgia can be challenging to diagnose due to its subjective symptoms, and healthcare-seeking behavior may influence the likelihood of receiving a diagnosis [9].
Socioeconomic Factors	Socioeconomic factors, including access to healthcare and socioeconomic status, may also play a role in the prevalence of fibromyalgia. Women’s health disparities and socioeconomic factors could contribute to differences in prevalence rates [10].

**Table 2:** Fibromyalgia Impact Questionnaire (FIQ-R) [15].

Section 1:	Physical Functioning
	1. Ability to do chores: How much does fibromyalgia affect your ability to do chores such as vacuuming, yard work, or laundry?
	2. Fatigue: How difficult has fatigue made it for you to perform everyday tasks?
	3. Sleep quality: How often has poor sleep affected your ability to do everyday tasks?
	4. Physical function: How much does fibromyalgia affect your ability to perform physical activities such as walking, climbing stairs, or lifting objects?
	5. Pain intensity: How severe is your pain on average over the last week?
	6. Muscle strength: How much has fibromyalgia affected your muscle strength?
	7. Balance: How much has fibromyalgia affected your balance?
**Section 2:**	**Overall Impact**
	8. Pain interference: How much does pain interfere with your daily life?
	9. Stiffness: How much does stiffness interfere with your daily activities?
	10. Depression: How often have you felt depressed because of your fibromyalgia symptoms?
	11. Anxiety: How often have you felt anxious because of your fibromyalgia symptoms?
	12. Memory problems: How often have you had difficulty remembering things because of your fibromyalgia symptoms?
**Section 3:**	**Symptom**
	13. Headaches: How often have you had headaches because of your fibromyalgia symptoms?
	14. Pain: How often have you had pain or tenderness all over your body because of your fibromyalgia symptoms?
	15. Tenderness: How often have you had pain or tenderness in your lower abdomen because of your fibromyalgia symptoms?
	16. Balance problems: How often have you felt dizzy or had problems with balance because of your fibromyalgia symptoms?
	17. Sensitivity to noise: How often have you felt bothered by noise because of your fibromyalgia symptoms?
	18. Sensitivity to light: How often have you felt bothered by bright lights because of your fibromyalgia symptoms?
	19. Sensitivity to odors: How often have you felt bothered by strong odors because of your fibromyalgia symptoms?
	20. Muscle weakness: How often have you felt that your muscles were too weak to do the things you wanted to do because of your fibromyalgia symptoms?
	21. Feeling of restlessness: How often have you had a sense of restlessness because of your fibromyalgia symptoms?
**Scoring:**	**Interpretation:**
	Each question is rated on a scale from 0 to 100 represents no impact and 10 represents maximum impact. The total score is the sum of all 21 responses, with a maximum possible score of 210. Higher scores indicate a greater impact of fibromyalgia on the individual’s life.
	- 0–39: Mild impact - 40–59: Moderate impact - 60 and above: Severe impact

**Table 3:** Demographic characteristics of the participants.

Characteristics	Healthy Controls	Fibromyalgia Syndrome
Gender (% female)	100%	100%
Age (years)	29.43 ± 6.4	38.85 ± 10.48
HADS-A	3.94 ± 1.7	13.54 ± 2.5 ***
HADS-D	1.5 ± 1.4	10.31 ± 3.4 ***
Pain severity (VAS ^#^)	0.71 ± 0.82	6.85 ± 1.91 **

^#^ VAS: Visual Analogical Scale, ** *p* < 0.001, and *** *p* < 0.0001.

## Data Availability

The raw data supporting the conclusions of this article will be made available by the authors on request.
